# Toll like receptor 4 (TLR4) gene polymorphism and its association with somatic cell score and milk production traits in Indian dromedary camels

**DOI:** 10.1080/10495398.2024.2331642

**Published:** 2024-03-23

**Authors:** Seema Bishnoi, Basanti Jyotsana, Virendra Kumar, Ved Prakash, Rakesh Ranjan, Shirish Dadarao Narnaware, Urmila Pannu

**Affiliations:** a CVAS, Rajasthan University of Veterinary & Animal Sciences, Bikaner, Rajasthan, India; b ICAR – National Research Centre on Camel, Bikaner, Rajasthan, India; c ICAR – Central Coastal Agricultural Research Institute, Ela, Old Goa, Goa, India

**Keywords:** Association, camel, gene, polymorphism, somatic cell score, TLR4, Milk production

## Abstract

Our study aimed to explore the genetic variation in the Toll-like receptor 4 (TLR4) gene and establish its association with somatic cell score (SCS) and milk production traits in four Indian camel breeds namely Bikaneri, Kachchhi, Jaisalmeri and Mewari. TLR4 gene fragment of 573 bp spanning 5’ UTR, exon-1 and partial intron-1 region was amplified and genotyped using the PCR-sequence based typing method. Only one SNP located at position C472T was identified. Genotyping revealed two alleles (C and T) and three genotypes: CC, CT and TT. The genotype frequencies for CC, CT and TT were 0.116, 0.326 and 0.558 and allele frequencies for C and T alleles were 0.279 and 0.721, respectively. Association study inferred that the effect of genotype on SCS, lactation yield (LY) and peak yield (PY) was non-significant however heterozygote (CT) genotypes recorded lower SCS and higher LY and PY. It can be concluded that the TLR4 gene possesses limited genetic variation, depicting polymorphism at a single locus in Indian camel breeds with a predominance of the TT genotype. The association study indicated that heterozygote animals possess better udder health and production performance, the statistical significance of which needs to be established using a large data set.

## Introduction

Since ancient times, domestic even-toed ungulates have played pivotal roles for man, being exploited for meat and milk, for fibre production, as beasts of burden for transport in agricultural/rural oriented communities; they were even worshiped.^1^ These animals have served traditional technologies since the very early era of domestication.^2^ Nowadays camels are increasingly being used as a dairy species due to the therapeutic value and health benefits of their milk. Like other camel countries demand for camel milk is also on the rise in India. Indian camel breeds are now being evaluated for their dairy potential and efforts to develop camel as a dairy species are underway.^3^ So, studying the genomic architecture of important candidate genes influencing udder health and milk production has the utmost significance.

Mastitis has been recognized as a major bottleneck in achieving milk production targets in livestock species. It is considered to be the most widespread and expensive disease plaguing the dairy industry across the globe.^4–6^ Like in other dairy animals, mastitis is an important health problem in lactating camels.^7^ The study by Ranjan et al.^8^ reported that the estimated cost of clinical mastitis to the tune of Indian Rs. 2179.26 (∼US$ 29.37) is a significant amount influencing the economics of camel dairying. Besides the financial implications of mastitis, its importance to public health should not be overlooked.^9^

The immune system consists of a complex network of cellular and non-cellular components, contributing equally to effective immune responses against pathogens. The Toll-like receptors (TLR) are a multigene family of pattern recognition receptors that are members of the TLR-Interleukin-1 superfamily. These receptors recognize a great variety of Pathogen Associated Molecular Patterns (PAMPs) and therefore play a central role in the initiation of an inflammatory response and subsequent adaptive immune response to microbial pathogens.^10^^,^^11^ TLRs are type-I transmembrane receptors structurally characterized by a cytoplasmic Toll/Interleukin-1 receptor (TIR) domain and by extracellular leucine-rich repeats (LRR), connected by a single transmembrane span.^12^ The TIR domain plays a crucial role in transmitting the signal to elicit inflammatory responses. TLR4 is a founding member of the TLR family, which responds to the ligand lipopolysaccharide (LPS), a component of the outer membrane of Gram-negative bacteria.^13^

The determination of gene polymorphism is important in farm animal breeding^14^^,^^15^ to define genotypes of animals and their associations with productive, reproductive and economic traits.^16–18^ TLR4 is a candidate gene for resistance to a large number of diseases.^19^ TLR4 plays an important role in pathogen recognition and subsequent initiation of the inflammatory and immune response, which makes it a suitable candidate gene for enhancing disease resistance in dairy cattle.^20^ TLR4 gene polymorphisms affect the susceptibility to infections, especially because of their location in the ligand recognition area of the receptor.^21^ In camels, TLR4 has been identified on the 4th chromosome, having 3 exons and 2 introns. Genomic studies have suggested that single nucleotide polymorphisms (SNPs) within the pattern recognition receptors (PRR) could lead to different responses to pathogens, and consequently result in mastitis resistance or susceptibility.^22^^,^^23^ Studies revealed that the TLR4 gene is upregulated in mastitis tissue in comparison with normal tissue.^23^^,^^24^

The somatic cell count (SCC) of milk is affected by various factors and is used as an indicator of mammary health on the basis that it reflects an immune response and thus the presence of infection.^25^ SCC of camel milk has not been studied extensively; as a result, the accepted ‘normal’ value of camel milk SCC has not been established. SCC in camel milk can be used to diagnose clinical mastitis or subclinical mastitis,^26^ and it was found that in dromedary milk, SCC decreases significantly with the stage of lactation.^27^ However, some researchers reported that the seasonal variation in milk quality indicators mainly SCC and total viable count in bulk camel milk is the consequence of seasonal reproduction and lactation curve characteristics of dromedaries.^28^ A strong association between TLR4 gene SNPs and somatic cell counts has been identified in Murrah buffaloes, suggesting that the TLR4 gene can be used as a molecular marker for mastitis resistance in buffaloes.^29^ The association of the TLR4 gene polymorphism with somatic cell count/score (SCC/SCS) may be attributed to the high genetic correlation between SCS and the occurrence of clinical mastitis.^30^ The studies in sheep, cattle and buffalo have reported an association between TLR4 polymorphisms and traits like milk yield, lactation persistency, milk fat and protein percentage, somatic cell score, and milk yield.^29^^,^^31^^,^^32^^,^^33^

Different genotyping methods have been explored in the past showing the growing interest in SNPs for association studies in livestock species.^34^ With the advancements in DNA sequencing technology, the polymerase chain reaction-sequence based typing (PCR-SBT) method is being preferred for genotyping by the researchers. PCR-SBT involves PCR amplification of specific fragments or regions of genes and sequencing of the amplicons. Compared with other SNP genotyping methods, it is particularly suited to identifying multiple SNPs in a small region, such as the highly polymorphic major histocompatibility complex region of the genome.^35^ The PCR-SBT method is advantageous over other typing methods in terms of accuracy, efficiency, and degree of automation.^36^

The TLRs of dromedary camels have not been characterized, except for TLR2.^37^ There is no report on the genetic variation of the TLR4 gene and its role in udder health and the production of Indian dromedary. Keeping in view the above facts, the present study attempts to characterize and explore genetic variation in the TLR4 gene and to find an association between the genetic variants and somatic cell score as well as certain milk production traits in Indian camels.

## Materials and methods

### Animals, blood sample and DNA extraction

The animals for the present study were selected from the herd of ICAR-NRCC, Bikaner. A total of 43 lactating she-camels (*Camelus dromedarius*) of 4 different breeds namely Bikaneri,^12^ Jaisalmeri,^11^ Mewari,^10^ and Kachchhi,^10^ were taken for the study. The selected animals were in the first to sixth parity of lactation. The average age of the first parity lactating camels was 4.81 years and for 5th and above parity was 15.94 years. Approximately 10 mL of venous blood was collected from the jugular vein of these lactating camels in sterile vacutainer tubes containing 0.5 M EDTA as an anticoagulant and were transported to the laboratory in the ice pack. In the laboratory, samples were kept in a deep freezer at −20 °C till the isolation of DNA. The genomic DNA was extracted from the blood by spin column method using a DNeasy blood and tissue kit (Qiagen, Germany). The quantity of isolated DNA was determined using Nanodrop spectrophotometer. Genomic DNA ran as a single band on agarose gel and the OD 260/280 ratio for all the samples was in range of 1.8–2.0, indicating good quality of extracted DNA. After checking the quantity DNA was stored at −20 °C for further use.

### Primer design and PCR amplification

Specific primers were designed (Table 1) using Primer3 software (http://frodo.wi.mit.edu/cgi-bin/primer3/primer3_www.cgi) based on the reference genome sequence available at NCBI: NW_011591011.1 (*Camelus dromedarius*). The primers used in this study are listed in Table 1. A TLR4 gene fragment of 573 bp size containing partial 5′ UTR, exon 1, and partial intron 1 region was amplified using polymerase chain reaction (PCR). For PCR amplification, the reaction mixture was kept in a programmable thermocycler (Applied Biosystems, USA). PCR was performed in a single tube containing ready to use 12.5 μL Go taq green master mix (Promega, USA), forward and reverse primers with a concentration of 10 pmol concentration each, genomic DNA with a concentration of 80–100 ng/μL and nuclease-free water (Promega, USA), which was added to make the total volume of reaction up to 25 μL. A gradient PCR program was used to find the appropriate annealing temperature (T_a_), which was further used to amplify the TLR4 gene fragment. The PCR cycling conditions were as follows: 95 °C for 4 minutes; 32 cycles of denaturation at 95 °C for 45 seconds; annealing at a specific temperature for 45 seconds; extension at 72 °C for 45 seconds; and final extension at 72 °C for 8 minutes. 5 µL of the amplified PCR product was run in 1.5% agarose gel electrophoresis for approximately 30 minutes in 1X TBE buffer along with a standard molecular marker (100 bp DNA ladder). The gel was visualized under the gel documentation system (Zenith Engineers, India). After amplification, the PCR product was stored at −20 °C for further analysis.

**Table 1.** Details of primers used for PCR amplification of TLR4 gene in Indian camel.

Primer name	Primer Sequence (5’-3’)	Length (bp)	Annealing temperature	Product size (bp)
TLR4 gene fragment XM_010998309.2	F – ATGCTTTCACAGGGCCACTT	20	57 °C	573 bp
	R – AAGCAAGGGCTTGAAGCAAAG	21		

### Sequence analysis, genetic polymorphism and phylogeny study

PCR products were purified using Wizard™ SV Gel and PCR Cleanup System (Promega, USA). A bidirectional sequencing of the purified PCR amplicons was performed by the Sanger dideoxy chain termination method (Biologia Research, India). A total of 43 PCR samples were sequenced, which included 5 animals of CC, 14 animals of CT, and 24 animals of TT genotypes. The forward and reverse sequences obtained for each animal were edited manually using Codon Code Aligner Software (USA) to generate different sequence patterns. The 573-bp sequence was assembled with the help of a reference sequence already available in the NCBI database. Gene (allele) and genotypic frequencies were calculated. To study the genetic variation in Indian camel TLR4 gene sequences and their relationship with different TLR4 gene sequence databases at the National Center for Biotechnology Information (NCBI), a pairwise and multiple sequence alignment was done using the BLAST software program (http://www.ncbi.nlm.nih.gov/). The phylogenetic tree was constructed for the estimation of the evolutionary relationship between different species using the neighbour joining method in the Molecular Evolutionary Genetics Analysis software, MEGA X.^34^ The evolutionary distances were computed in units of the number of nucleotide substitutions per site using the Poisson correction method.^34^

### Milk somatic cell counting

Fresh milk samples were collected from 43 she-camels selected for the study. The somatic cell count (SCC) of collected milk samples was done as per the method described by Schalm et al.^38^ The somatic cell count (SCC) was log (base 10) transformed into somatic cell score (SCS) for further analysis. The SCC and log SCC are presented in Table 4.

### Milk production data recording

The lactating she-camels were reared under a semi-intensive system of management. All the animals under study were subjected to similar environmental conditions of temperature and humidity. The lactating camels were housed in a group pen. Evening milking was done after 8–10 hrs intervals. Hand milking was followed and trained members of the traditional *Raika* community were engaged in milking. The milk recording commenced on the 15th day after calving. Till the weaning age of three months weekly test day milk recording was done and thereafter daily milk yield recording was done. The details of feeding, grazing and milking management followed at the farm have been reported by Prakash et al.^3^ The morning and evening milk yield was recorded in kg using a digital weighing balance. Daily milk yield was calculated as the sum of morning and evening milk yield. The lactation yield (LY) was calculated as the sum of the total daily milk in the lactation period. The highest recorded daily milk yield during lactation was considered as peak yield. Peak milk yield proved to be a good predictor of lactation yield. Peak milk yield has been found significantly correlated (r = 0.91) with lactation yield.^39^ The higher correlation between 305-day milk yield and peak yield compared to decay as an indicator for lactation persistency indicates that peak yield is more important in determining the lactation yield and can be used as a management tool to monitor milk production performance of the herd.^40–42^

### Genetic association study

The association study of TLR4 genotypes with milk somatic cell score (SCS), Lactation Yield (LY), and peak milk yield (PY) was performed using least squares method of the General Linear Model (GLM) procedure of the SPSS 20 software.

### Genetic association with milk somatic cell count

A mixed model equation was used for studying the association of TLR4 genotype with log SCC (SCS).

$$Y_{\text{ijklm}}=\mu +{\text{G}}_{\text{i}}+{\text{H}}_{\text{j}}+{\text{P}}_{\text{k}}+{\text{Y}}_{\text{l}}+{\text{bS}}_{m}+e_{\text{ijklm}}$$

Where, Y_ijklm_ is the log SCC (SCS) value of an animal of ith genotype, jth breed, kth parity, lth year of calving. μ is the overall mean, and G_i,_ H_j,_ P_k_ and Y_l_ are fixed effects of genotype, breed, parity and calving year, respectively. bS_m_ is the regression of lactation stage (days in milk) on the day of sample collection for somatic cell counting and e_ijklm_ is random error.

### Genetic association with milk production traits

For the association of genotype with lactation yield and peak milk yield following fix effect model was used.

$$Y_{\text{ijklm}}=\mu +{\text{G}}_{\text{i}}+{\text{H}}_{\text{j}}+{\text{P}}_{\text{k}}+{\text{Y}}_{\text{l}}+e_{\text{ijklm}}$$

Where, Y_ijklm_ is the lactation yield/peak milk yield of an animal of ith genotype, jth breed, kth parity, and lth year of calving. μ is the overall mean, G_i,_ H_j,_ P_k_ and Y_l_ are fixed effects of genotype, breed, parity and calving year, respectively and e_ijklm_ is random error.

## Results and discussion

### Genetic characterization and polymorphism

**Figure 1.**
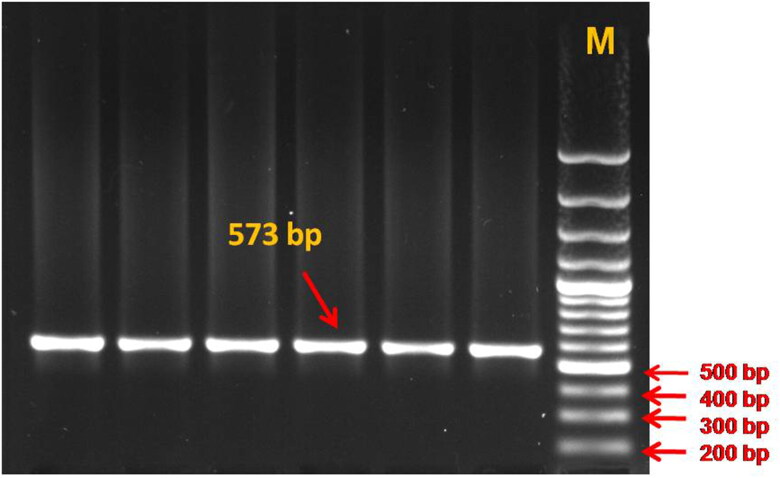
PCR amplification of TLR4 gene fragment spanning 5’ UTR, exon 1 and partial intron 1 region (573 bp), resolved on 1.5% agarose gel (M – Molecular weight marker = 100 bp).

**Figure 2.**
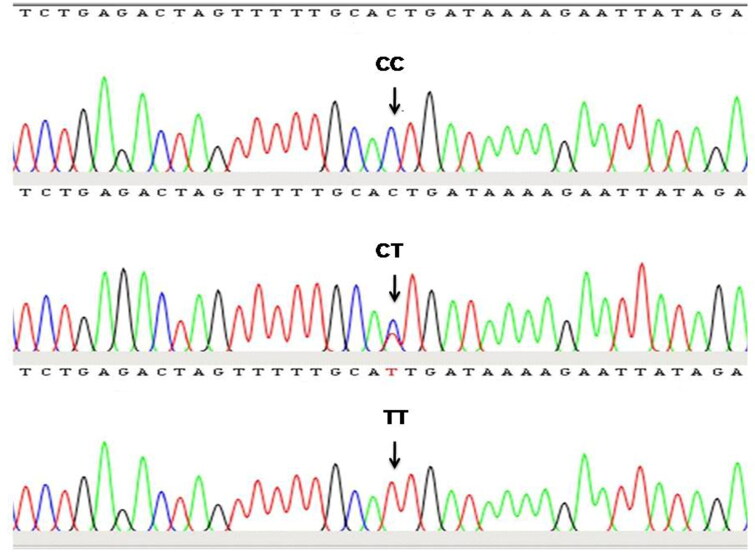
Sequence chromatogram of different genotypes at C472T SNP locus identified in intron-1 region of TLR4 gene.

To the best of our knowledge, this is the first report in which genetic variation in the TLR4 gene and its association with somatic cell score and milk production traits in dromedary camels has been studied. The gel electrophoresis of the PCR product confirmed amplification of the target gene in the form of a clear band of expected size (Figure 1). The assembly and editing of the forward and reverse sequences confirmed the amplification of the TLR4 gene region of 573 bp size, spanning 5′ UTR, exon-1, and partial intron-1. The sequence analysis revealed only one SNP located in the intron-1 region of the TLR4 gene at the 472nd position (C472T) (Figure 2). The TLR4 gene sequences were assembled with the help of a reference sequence and three genetic variants were obtained which were submitted with accession numbers OM621865, OM621866, and OM621867 to the NCBI Gen Bank.

Contrary to the reports of high genetic polymorphism in other livestock species,^20^^,^^29^^,^^32^ we found no SNPs in the exonic region and only one SNP at the intron-1 region. However, our findings were similar to studies in bovines, pigs and ovines which also reported polymorphism in the intronic regions.^32^^,^^43^^,^^44^ Yang et al.^45^ suggested the role of intronic variants in regulating gene expression. The introns also play a pivotal role in mRNA export, transcription coupling, splicing etc.^46^ Absence of polymorphism in the studied coding region of the camelid’s TLR4 gene, depicted the unique genomic architecture suggesting either possible selective pressure being exerted on the gene, and/or the role of other innate immune system components in camelids’ evolution and adaptation. Researchers have also reported a low level of polymorphism in Major Histocompatibility Complex class II genes in old-world camels as compared to other mammalian species.^47^^,^^48^

Two alleles (T and C) and three genotypes (TT, CT and CC) were detected in our study. The genotypic frequency of the studied population was found to be 0.558, 0.326 and 0.116 for TT, CT and CC genotypes, respectively, whereas the gene frequency of T and C allele was found to be 0.721 and 0.279, respectively (Table 2). The studied population were in Hardy-Weinberg equilibrium (*P* = 0·211).

**Table 2.** Genotype and allele frequency of the studied TLR4 gene fragment in Indian Dromedary camel.

Animals Studied (*N* = 43)	Genotypes			Chi-square (χ^2^) value	*P*-value
	TT	CT	CC	1.56	0.211
Total Genotypes	24	14	5		
Genotype Frequency	0.558	0.326	0.116		
Allele Frequency	*T* = 0.721		*C* = 0.279		

**Figure 3.**
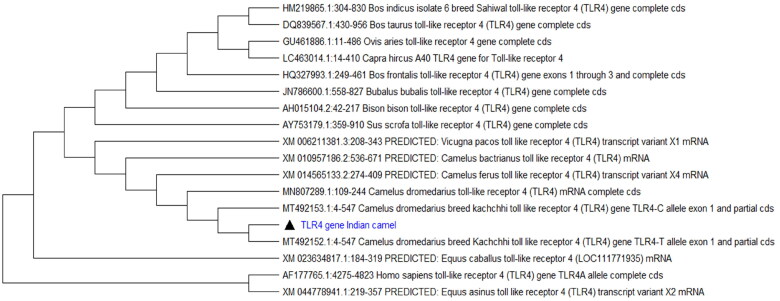
Phylogenetic tree constructed using Neighbor-Joining method.

The phylogenetic tree constructed based on the TLR4 nucleotide sequences showed camelids formed a separate cluster (Figure 3), showing their uniqueness with other livestock species. The camelid species formed a clade with Vicugna, *Camelus bactrianus*, and *Camelus ferus*, showing closer similarity. Phylogenetic analysis also showed dissimilarity between camels and other livestock species. The Indian dromedary camels showed very high sequence identity with other dromedaries, ferus, and Bactrian camels, which ranged from 99.72 to 100%. Among the domesticated species the highest sequence identity of 88.24, 87.77, and 81.92%, respectively, was observed between horse, ass, and pig, thus horse being the closest to the camelid family (Table 3). These findings were in accordance with the previous study, which showed that homology in Toll-like receptors exists in all vertebrates that share some conserved regions.^49^

**Table 3.** Comparison of nucleotide sequence of Indian Dromedary Camel TLR4 gene (Accession No. OM621865) with other species.

Species	Accession number	% identity
Arabian Camel	MT492152.1	100
Camel Dromedary	MN807289.1	100
Arabian Camel	MT492153.1	99.82
Camelus Ferus	XM_014565133.2	99.27
Camel Bactrian	XM_010957186.2	99.27
Alpaca	XM_006211381.3	97.06
Horse	XM_023634817.1	88.24
Ass	XM_044778941.1	87.77
Pig	AY753179.1	81.92
Bos Frontalis	HQ327993.1	79.13
Bison	AH015104.2	78.53
Buffalo	JN786600.1	77.93
Goat	LC463014.1	75.88
Zebu Cattle	HM219865.1	75.48
Exotic Cattle	DQ839567.1	75.26
Sheep	GU461886.1	74.38
Human	AF177765.1	70.34

### Association study

**Figure 4.**
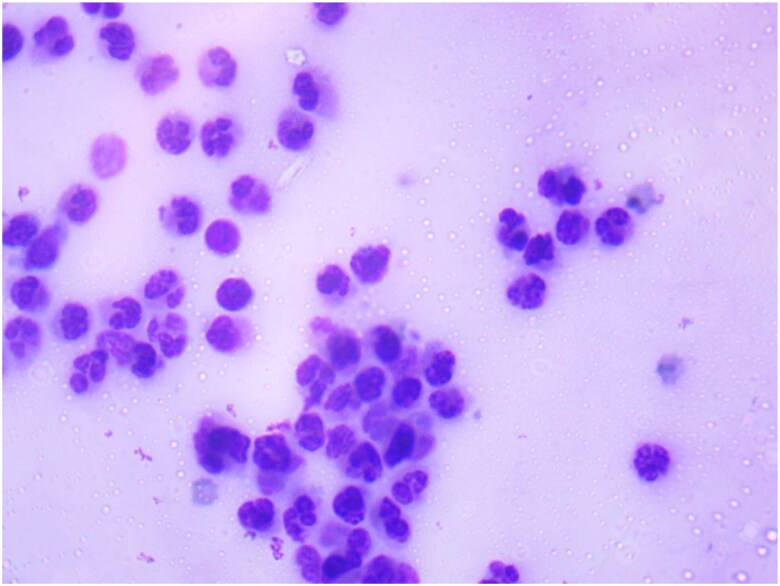
Microscopic examination of milk somatic cells smear stained with Newman stain showing somatic cells (numerous milk somatic cells present in milk smear).

The milk sample smear was counted for somatic cells and the somatic cell count (SCC) was log-transformed (base 10) into somatic cell score (SCS) for further analysis in our study (Figure 4). SCS was used in our study rather than SCC due to its higher heritability and thus more suitable for genetic analysis studies. Monardes et al.^50^ found that the heritability of SCC was around 6% and the heritability of SCS was 12%. The somatic cell count of the studied population ranged from about 17.8 × 10^3^ to 922.6 × 10^3^ cells ml^−1^. The average SCC was 218.3 × 10^3^ cells ml^−1^. Nagy et al.^28^ studied somatic cell count in the bulk milk of dromedary camel and found the geometric means of SCC to be 394 × 10^3^ cells/ml. An increase in the somatic cell count (SCC) of more than 5x10^5^ cells/ml is considered to be an indicator of udder infection in cattle as well as camels.^51^^,^^52^ The upper limit value in the present study was higher than the cut-off value but the average of the somatic cell count of the present study was lower than the cut-off value i.e., 5x10^5^ cells/ml depicting the better udder health status of the studied population. Somatic cells are a part of the natural defence mechanism and are therefore a reflection of the inflammatory response to an intramammary infection or triggers of the immune system.

The association of SCC has been explored much in bovine species however information on SCC of camel milk is limited. The means with standard errors for SCS for different genotypes and other factors are presented in Table 5. The SCS did not differ significantly between genotypes, breed, parity, lactation stage and calving year. Several factors like breed of the animal, age, parity, season of the year, stage of lactation, milk yield potential and husbandry practices can modulate the risk of clinical mastitis in camels.^8^^,^^53–55^

**Table 4.** Somatic cell count and somatic cell score data recorded in the population with mean values.

	Minimum	Maximum	Mean	Std. Deviation
Somatic cell count (SCC)	17858	922638	218352.51 ± 29797.29	195393.86
Somatic cell score (log SCC)	4.25	5.96	5.19 ± 0.06	0.3797

**Table 5.** Least squares means of somatic cell score, lactation yield and peak yield in Indian Dromedary Camel.

	No. of observations	Log SCC	Lactation yield (kg)	Peak Milk Yield (kg)
Overall LSM	43	5.29 ± 0.08	1839.97 ± 184.11	6.95 ± 0.37
Genotype		NS	NS	NS
CC	5	5.45 ± 0.17	1888.56 ± 414.03	6.67 ± 0.83
CT	14	5.19 ± 0.10	2141.59 ± 252.25	7.82 ± 0.50
TT	24	5.23 ± 0.09	1489.75 ± 206.47	6.37 ± 0.41
Breeds		NS	NS	NS
Bikaneri	12	5.36 ± 0.14	1649.67 ± 320.58	6.52 ± 0.64
Jaisalmeri	11	5.51 ± 0.14	2093.64 ± 319.20	7.09 ± 0.64
Kachchhi	10	5.00 ± 0.12	1947.07 ± 300.47	7.89 ± 0.60
Mewari	10	5.29 ± 0.13	1669.55 ± 310.05	6.33 ± 0.62
Parity		NS	NS	*
1	4	5.16 ± 0.19	1029.14 ± 459.92	4.54 ± 0.92
2	17	5.12 ± 0.09	2068.87 ± 229.55	7.14 ± 0.46
3	11	5.37 ± 0.13	2024.92 ± 302.03	7.56 ± 0.60
4	6	5.46 ± 0.15	2294.12 ± 356.38	8.41 ± 0.71
≥5	5	5.34 ± 0.17	1782.78 ± 413.54	7.12 ± 0.83
Calving year		NS	NS	NS
2020	17	5.47 ± 0.16	2021.20 ± 265.75	6.71 ± 0.53
2021	26	5.11 ± 0.12	1658.73 ± 193.01	7.20 ± 0.39
Lactation stage		NS	-	-
		(R^2^ = 0.43)	(R^2^ = 0.29)	(R^2^ = 0.49)

NS – non-significant, * – significant (*p* < 0.05), ** – highly significant (*p* < 0.01).

The difference in SCS value among the genotypes CC, CT and TT of the screened population was found to be non-significant. However, heterozygous animals (CT genotype) recorded lower somatic cell score (SCS) than homozygotes. Sharma et al.^20^ found a significant association of TLR4 haplotype GCC (54%) with lower somatic cell scores in Canadian Holstein cattle. Our result was in contrast to Wang et al.^43^ who explored the TLR4 genetic variants and associated homozygous genotype AA with a lower somatic cell score (SCS) in bovines. Mesquita et al.^22^ found that combined genotypes AACCCC, GGTCGG and GACCGC of the TLR4 gene presented lowest SCS in Brazilian Holstein cows, suggesting that the alleles A and C could be associated with genotypes of lower SCS. Noori et al.^33^ also reported significantly lower SCS in homozygote CC genotypes of the TLR4 gene compared to the TT genotype in dairy cattle. In our study, the effect of genotypes was found non-significant on lactation yield (LY) as well as peak yield (PY). However, LY and PY were found higher in the CT genotype as compared to (CC and TT) homozygotes (Table 5). These findings indicated better udder health status of heterozygote animals compared to homozygotes, making them more productive. Noori et al.^33^ revealed contrasting results with the homozygous genotype of TLR4 gene, which was associated with higher 305-day milk yield and breeding value for milk yield in Iranian Holstein cattle. Sharma et al.^20^ also found a significant association of TLR4 haplotype GCC (54%) with higher lactation persistency in Canadian Holstein cattle, showing contrasting findings from our study. Pasandideh et al.^56^ reported that the AC and CC genotypes in PPARGC1A and OPN loci are associated with the highest mean SCC in Iranian Holstein cattle.

Besides genotype, the effect of breed was found non-significant on SCS, LY and PY. The Kachchhi breed recorded highest peak yield (7.89 ± 0.60 kg) and lowest SCS. Prakash et al.^3^ also reported the Kachchhi breed as the highest milk producer among Indian breeds. The breed-related differences for mastitis have been reported in Indian Dromedary camel.^8^^,^^57^ The breed-related differences may be due to differences in production potential^58^ or differences in their genetic makeup.^56^ The effect of parity was significant on PY (*p* < 0.05) and non-significant on LY and SCS. The third and fourth parity animals have comparatively higher SCS. Ranjan et al.^8^ also reported a higher incidence of mastitis in third and fourth-parity Indian dromedary camels. LY and PY were found to be highest in 4th parity (2294.12 ± 356.38 kg and 8.41 ± 0.71 kg, respectively). Animals with high milk yield potential appeared more susceptible to clinical mastitis.^8^ Higher milk yield in the fourth parity is consistent with the report of Bekele et al.^59^ and Prakash et al.^3^

## Conclusion

This is the first report in camel where TLR4 gene polymorphism was studied and associated with somatic cell score and lactation performance of the animals. The study revealed close similarity of the Indian dromedary TLR4 gene sequence with other camelid sequences. Further, the TLR4 gene in the Indian camel population showed no polymorphism in the exonic region and only one SNP at locus C472T in the intron-1 region was found. Three TLR4 genotypes TT, CT, and CC with frequencies 0.558, 0.326 and 0.116, respectively were detected.

The association study revealed a non-significant effect of genotypes on somatic cell score, lactation yield, and peak yield. However, heterozygous genotypes recorded lower somatic cell scores and higher milk yield than homozygotes genotypes in the population, indicating their better udder health and production status. We were able to find a positive trend between TLR4 heterozygous genotype, lactation performance, and somatic cell score, but it may not be reliable to conclude the role of TLR4 genotypes in milk yield and health status of the animals since the number of animals in each genotype was very small and the results were also not significant. Despite the limitations, the findings and important insights of the present work can contribute to studies exploring camel unique immune status and its role in production and disease. Further, studies to screen the identified SNP in a large, diversified population are suggested to establish it as a marker for determining the udder health and milk production of dromedary camels.
